# Biofilm Removal and Bacterial Reduction Using a Low-Cost Ultrasonic Bath Sonicator with Povidone–Iodine on Polyethylene: An In Vitro Periprosthetic Joint Infection Model

**DOI:** 10.3390/antibiotics15080810

**Published:** 2026-08-19

**Authors:** Bhuwad Chinwatanawongwan, Chavarat Jarungvittayakon, Siwadol Wongsak, Paphon Sa-ngasoongsong, Pattarana Sae-Chew, Thidarat Rujirawat, Penpan Payattikul

**Affiliations:** 1 Department of Orthopaedics, Faculty of Medicine Ramathibodi Hospital, Mahidol University, Bangkok 10400, Thailand; bhuwadc@nu.ac.th (B.C.); siwadolrama@hotmail.com (S.W.); paphonortho@gmail.com (P.S.-n.); 2 Department of Orthopaedics, Faculty of Medicine, Naresuan University, Phitsanulok 65000, Thailand; 3 Office of Health Science Research, Faculty of Medicine Ramathibodi Hospital, Mahidol University, Bangkok 10400, Thailand; pattarana.sae@mahidol.ac.th (P.S.-C.); thidarat.ruj@mahidol.ac.th (T.R.); payattikul@yahoo.com (P.P.)

**Keywords:** periprosthetic joint infection, ultrasonic bath sonicator, biofilm, *Staphylococcus epidermidis*, povidone–iodine

## Abstract

**Background:** Biofilm formation on polyethylene surfaces contributes significantly to treatment failure in periprosthetic joint infection (PJI). In certain clinical situations, such as discontinued implant systems, replacement polyethylene liners may be unavailable, necessitating more complex revision procedures. This study evaluated the efficacy of a low-cost ultrasonic bath sonicator for biofilm removal and bacterial reduction on polyethylene surfaces, with and without povidone–iodine. **Methods:** An exploratory in vitro experimental study was performed using twenty ultra-high-molecular-weight polyethylene (UHMWPE) coupons (1 × 1 cm, 3 mm thickness) allocated into five groups (*n* = 4/group): negative control, positive control, povidone–iodine only (PVP-I), sonication only (SON), and sonication with povidone–iodine (SON+PVP-I). Biofilm formation using *Staphylococcus epidermidis* was established through an optimized protocol developed from sequential pilot experiments. Residual biofilm was quantified using optical density at 600 nm (OD600), while viable bacterial burden was determined by colony-forming unit counts per milliliter (CFU/mL). **Results:** SON demonstrated the lowest residual biofilm burden (OD600 = 0.194), whereas no culturable bacteria were detected above the assay detection limit (<10 CFU/mL) in the SON+PVP-I group. SON alone resulted in the greatest reduction in residual biofilm biomass, while the addition of PVP-I was associated with reduced recovery of culturable bacteria. **Conclusions:** A low-cost ultrasonic bath sonicator effectively reduced residual biofilm biomass on polyethylene surfaces, while no culturable bacteria were detected following combined sonication and povidone–iodine treatment. These findings suggest that sonication and chemical disinfection may provide complementary roles in polyethylene decontamination strategies. However, because of the exploratory study design and non-equivalent bacterial recovery procedures across treatment groups, the bacterial recovery findings should be interpreted cautiously. Further studies are required before clinical application.

## 1. Introduction

Periprosthetic joint infection (PJI) remains one of the most devastating complications following total joint arthroplasty and continues to impose substantial clinical and economic burdens. The incidence of PJI is approximately 1–2% after primary arthroplasty and increases further following revision procedures. Despite advances in surgical techniques and antimicrobial therapies, successful eradication of infection remains challenging because of bacterial biofilm formation on implant surfaces [1,2,3].

Biofilm is a structured microbial community enclosed within a self-produced extracellular polymeric substance (EPS) matrix consisting of proteins, polysaccharides, extracellular DNA, and lipids. The EPS matrix protects bacteria from host immune responses and limits antimicrobial penetration, thereby increasing bacterial persistence and treatment failure [4,5,6].

For acute hematogenous or early postoperative PJI, debridement, antibiotics, and implant retention (DAIR) remains a commonly utilized treatment strategy. A critical component of DAIR is modular polyethylene exchange to reduce bacterial burden and remove biofilm-contaminated surfaces. However, in some situations, such as discontinued implant systems or unavailable modular polyethylene components, isolated liner exchange may not be possible. Consequently, complete implant system conversion may become necessary, increasing surgical complexity, morbidity, and bone loss [7].

Various approaches have been investigated to improve implant decontamination and biofilm removal. Ultrasound has emerged as a potential approach because acoustic cavitation generated by ultrasonic waves can produce localized mechanical forces capable of detaching microorganisms from implant surfaces [8,9]. Sonication has also been widely used in the diagnosis of PJI, improving bacterial recovery by releasing microorganisms embedded within biofilms. Trampuz et al. demonstrated superior sensitivity of sonicate-fluid culture compared with conventional periprosthetic tissue culture for the diagnosis of PJI [10].

Previous studies have also investigated ultrasound as a method for biofilm removal. Russo et al. reported that piezoelectric ultrasonic debridement significantly reduced bacterial burden on orthopedic implant materials [11]. Similarly, Wu et al. demonstrated substantial biofilm reduction using direct-contact low-frequency ultrasound [12]. However, these approaches used specialized ultrasonic systems, and evidence regarding the use of commercially available bath-type ultrasonic devices for biofilm removal from polyethylene surfaces remains limited.

*Staphylococcus epidermidis*, one of the common pathogens implicated in PJI, exhibits strong adhesion and biofilm-forming capacity on polyethylene surfaces [13]. In addition to mechanical biofilm removal, povidone–iodine has demonstrated antimicrobial and antibiofilm activity and may therefore provide an adjunctive effect when combined with sonication [14].

Compared with probe-based or direct-contact systems, bath-type sonication provides non-contact treatment and broad surface coverage with less operator dependency. Commercially available ultrasonic bath devices are also relatively inexpensive and readily accessible. Therefore, this study focused on evaluating the feasibility of a low-cost, consumer-grade bath-type ultrasonic device for biofilm removal from UHMWPE surfaces, with povidone–iodine evaluated as an adjunct for reducing viable bacterial recovery.

Furthermore, previous investigations have used varying biofilm culture conditions and incubation periods. Prior to the main experiment, sequential pilot experiments were therefore conducted to establish a consistent mature biofilm model on ultra-high-molecular-weight polyethylene (UHMWPE) surfaces.

The primary objective of this study was to evaluate the effectiveness of sonication with and without povidone–iodine in reducing residual biofilm biomass on UHMWPE surfaces. The secondary objective was to evaluate viable culturable bacterial recovery following each intervention.

## 2. Materials and Methods

### 2.1. Study Design

This in vitro experimental study evaluated the effectiveness of a low-cost ultrasonic bath sonicator with and without povidone–iodine (PVP-I) for reducing residual biofilm biomass and culturable bacterial burden on ultra-high-molecular-weight polyethylene (UHMWPE) surfaces.

Twenty UHMWPE coupons measuring 1 × 1 cm with a thickness of 3 mm were used in the main experiment and allocated into five experimental groups (*n* = 4 per group): negative control (CTRL−), positive control (CTRL+), povidone–iodine treatment alone (PVP-I), sonication in normal saline (SON), and sonication in povidone–iodine (SON+PVP-I). The detailed treatment conditions for each group are described in Section 2.3.

The study was conducted as an exploratory proof-of-concept experiment. The sample size was determined based on the feasibility of the experimental protocol.

### 2.2. Biofilm Model Optimization

**Figure 1:**
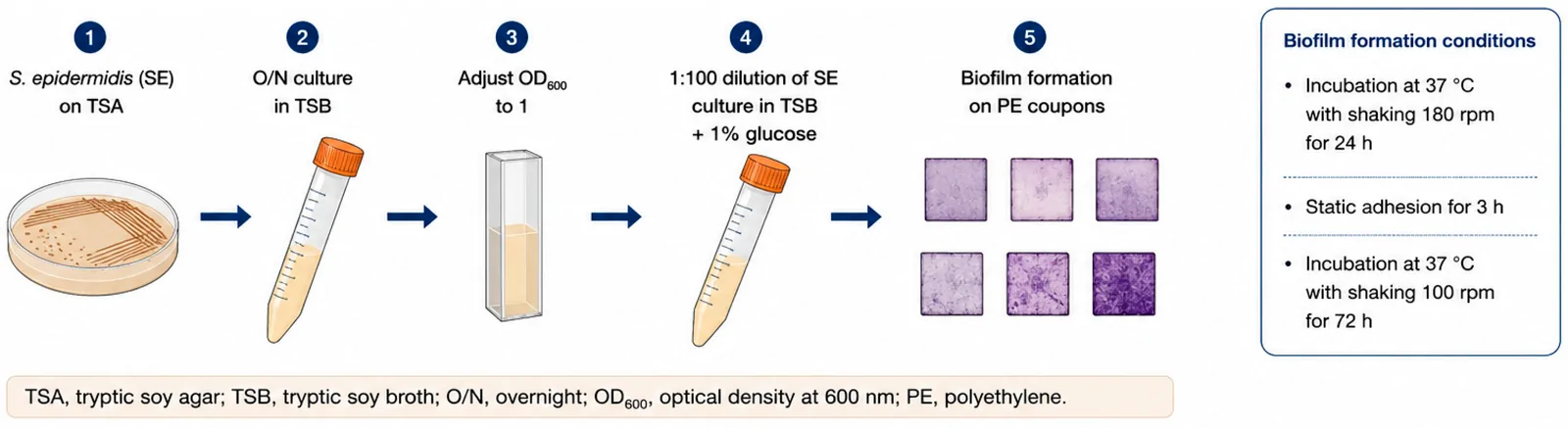

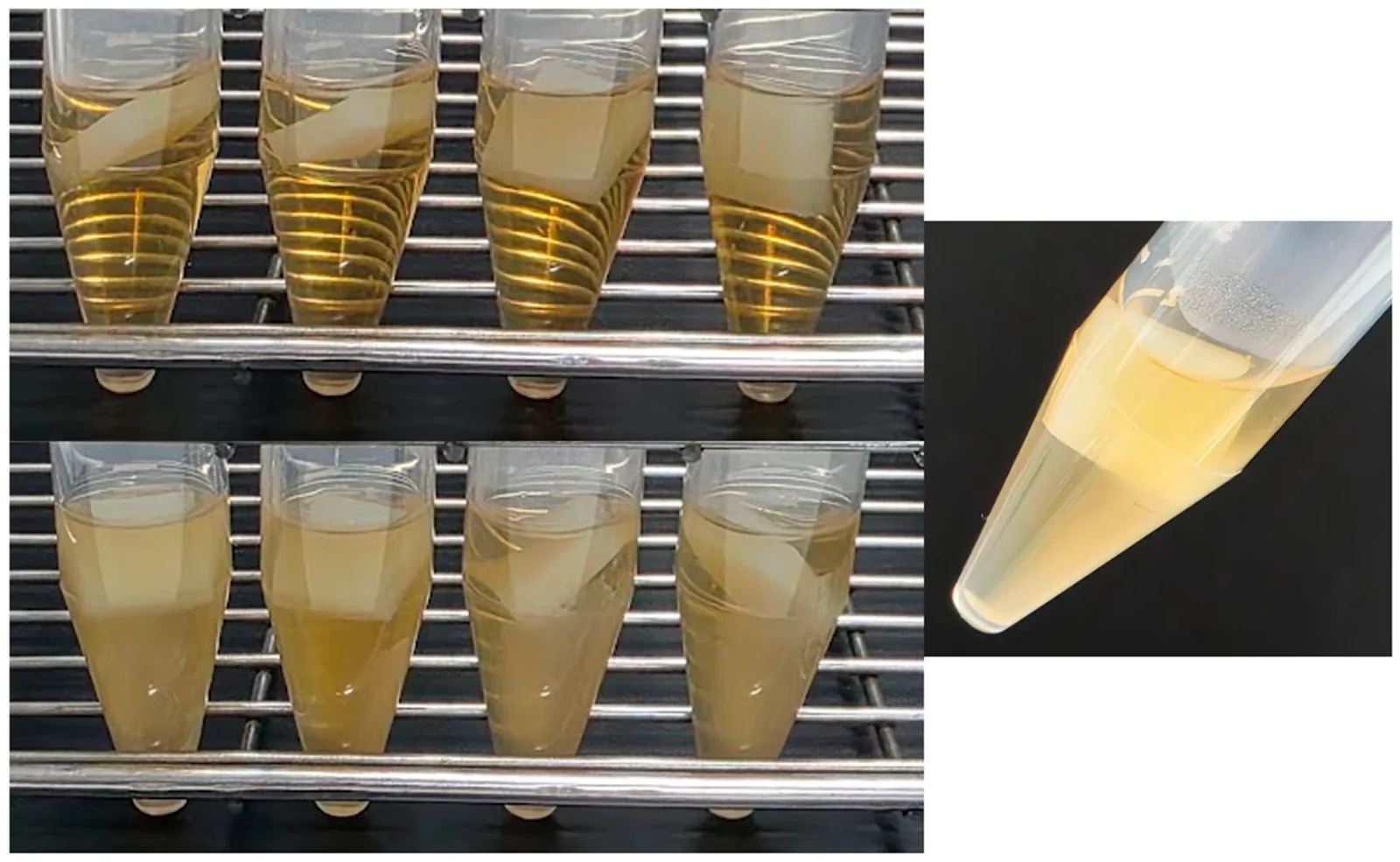
Schematic representation of the optimized *Staphylococcus epidermidis* biofilm formation protocol on UHMWPE coupons.

Prior to the main experiment, eight pilot experiments were performed to optimize biofilm formation on UHMWPE coupons (Figure 1). The optimization process evaluated incubation duration, static and shaking conditions, staged bacterial growth and adhesion, background crystal violet staining, and bacterial recovery following sonication. Substantial variability in background crystal violet staining among UHMWPE coupons was observed during the pilot experiments. Based on these observations, a background crystal violet staining threshold (OD600 < 0.5) was established for coupon selection in the main experiment.

The pilot experiments also evaluated different biofilm maturation periods. Based on the overall pilot findings, a staged protocol consisting of an initial bacterial growth phase, a static adhesion period, and subsequent biofilm maturation under shaking conditions was selected. A 72-h maturation period was selected for the main experiment because it provided recoverable viable bacteria following sonication with relatively low residual crystal violet staining compared with the longer maturation periods evaluated. A summary of the eight pilot optimization experiments is provided in Table 1.

For the main experiment, *Staphylococcus epidermidis* was cultured overnight in tryptic soy broth (TSB). The bacterial suspension was adjusted to an optical density at 600 nm (OD600) of 1.0 and subsequently diluted 1:100 in TSB supplemented with 1% glucose. UHMWPE coupons were incubated with 2 mL of the bacterial suspension at 37 °C with shaking at 180 rpm for 24 h, followed by a 3-h static adhesion period. The coupons were then incubated at 37 °C with shaking at 100 rpm for an additional 72 h to allow biofilm maturation.

### 2.3. Experimental Intervention

**Figure 2:**
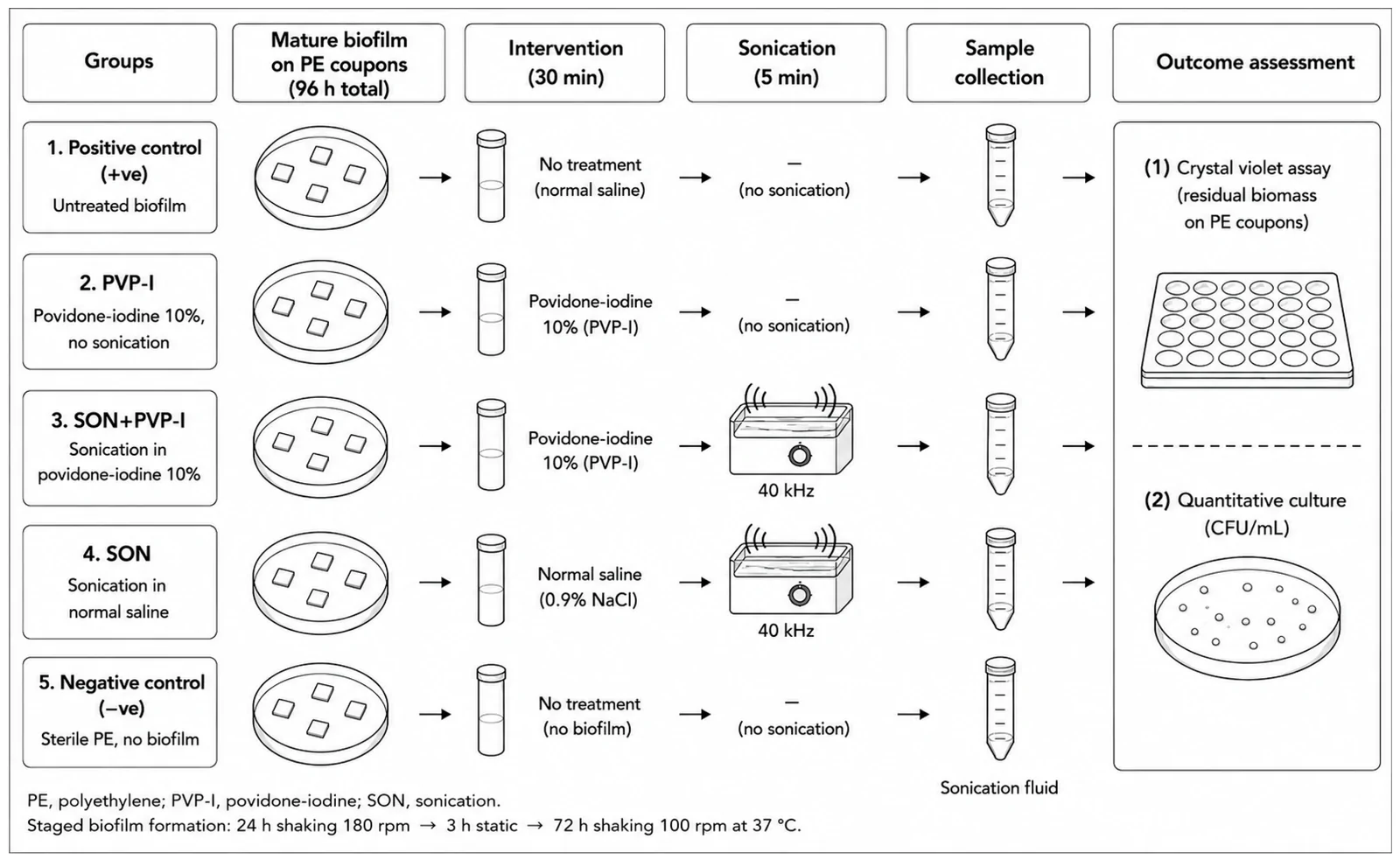
Schematic illustration of the experimental intervention and outcome assessment.

For the main experiment, 48 newly prepared UHMWPE coupons were screened for background crystal violet staining before bacterial inoculation (Figure 2). Coupons with background OD600 values <0.5 were considered eligible for inclusion. Of the 48 coupons screened, 22 met the predefined criterion. Twenty coupons were selected for the main experiment and randomly allocated to five experimental groups (*n* = 4 per group), whereas the remaining two eligible coupons were not required for the study. The selected coupons were allocated to five experimental groups: positive control (CTRL+), povidone–iodine treatment (PVP-I), sonication in normal saline (SON), combined sonication and povidone–iodine treatment (SON+PVP-I), and negative control (CTRL−).

The CTRL+ group received no treatment and served as the untreated biofilm control. In the PVP-I group, each coupon was placed individually in a sealed well containing 2 mL of 10% povidone–iodine solution for 10 min without sonication. In the SON group, each coupon was placed individually in a sealed well containing 2 mL of sterile normal saline and subjected to continuous ultrasonic bath sonication for 10 min. In the SON+PVP-I group, each coupon was placed individually in a sealed well containing 2 mL of 10% povidone–iodine solution and subjected to continuous ultrasonic bath sonication for 10 min. The CTRL− group consisted of sterile UHMWPE coupons without bacterial inoculation or treatment and was included to assess background crystal violet staining.

For the SON and SON+PVP-I groups, the sealed sample wells were placed in the water-filled ultrasonic bath, allowing indirect transmission of ultrasonic energy through the well walls to the treatment solution. Sonication was performed using a 2-L digital ultrasonic bath cleaner (model MH-010S; purchased from FASAI TOOL, Samut Sakhon, Thailand; manufacturer not specified) operating at a frequency of 40 kHz with a rated device ultrasonic power of 60 W and a single ultrasonic transducer. Sonication was applied continuously for 10 min. All procedures were initiated at room temperature without activation of the device’s built-in heating function. The temperature during sonication was not continuously monitored.

The treatment duration was standardized to 10 min across the PVP-I, SON, and SON+PVP-I groups. Following the assigned interventions, the coupons were processed for assessment of residual biofilm biomass and viable bacterial recovery, as described below.

### 2.4. Outcome Measurements

Primary outcome: Residual biofilm biomass

The primary outcome was residual adherent biofilm biomass following the assigned intervention. Residual biofilm biomass was quantified using the crystal violet assay, which measures total surface-associated biomass rather than bacterial viability [15].

Following the intervention, the coupons were washed three times with phosphate-buffered saline (PBS) to remove non-adherent material and stained with crystal violet solution for 10 min at room temperature. Excess stain was removed by washing with distilled water. The crystal violet retained by the residual adherent biomass was subsequently solubilized using 30% glacial acetic acid, and the optical density was measured at 600 nm (OD600). Lower OD600 values indicated lower amounts of residual crystal violet-stained biomass.

Secondary outcome: Viable bacterial quantification

The secondary outcome was the recovery of viable culturable bacteria following the assigned intervention. Bacterial suspensions obtained during the experimental procedures were serially diluted and plated on tryptic soy agar (TSA) for colony enumeration. In groups not undergoing sonication as part of the initial intervention, an additional sonication step was used to facilitate the recovery of bacteria from the coupon surface before culture. In the SON group, bacterial recovery was assessed following the initial treatment sonication and again following a subsequent sonication step to evaluate additional culturable bacteria recoverable after the first sonication. The resulting suspensions were serially diluted and cultured for colony enumeration.

Bacterial counts were expressed as colony-forming units per milliliter (CFU/mL) and transformed to log10 CFU/mL where appropriate. For samples with low bacterial counts, 100 µL of undiluted suspension was plated. Based on a minimum detectable count of one colony, the limit of detection of the culture assay was 10 CFU/mL. Samples with no colony growth were therefore reported as below the detection limit (<10 CFU/mL) rather than as evidence of complete bacterial eradication.

Because the bacterial recovery procedures were not fully equivalent across all intervention groups, CFU measurements were interpreted as culturable bacterial recovery under the respective treatment workflows rather than as a standardized quantitative measure of the total residual viable bacterial burden on the coupon surface. Accordingly, direct quantitative comparisons of CFU values between groups were interpreted with caution.

Statistical Analysis

Continuous variables were summarized as mean ± standard deviation (SD) and median with interquartile range (IQR). Differences in residual biofilm biomass (OD600) among the five experimental groups were explored using the Kruskal–Wallis test. When the overall test was significant, exact two-sided Mann–Whitney U tests were performed for pairwise comparisons, with Holm adjustment for multiple comparisons. Given the exploratory study design and small sample size (*n* = 4 per group), the inferential analyses were interpreted cautiously. Because bacterial recovery procedures were not equivalent across all intervention groups, CFU measurements were analyzed descriptively and were not subjected to direct between-group inferential testing. Statistical significance was defined as a two-sided adjusted *p*-value < 0.05.

## 3. Results

### 3.1. Residual Biofilm Biomass

Eight pilot optimization experiments were conducted before the main experiment to establish the biofilm formation protocol. The sequential modifications and rationale for selecting the final protocol are summarized in Table 1. Based on the overall pilot findings, a staged protocol consisting of 24 h of shaking incubation at 180 rpm, followed by 3 h of static adhesion and 72 h of maturation at 100 rpm, was selected for the main experiment.

Residual biofilm biomass, as assessed by crystal violet staining, differed among the experimental groups. The positive control (CTRL+) group demonstrated the highest OD600 value (3.098 ± 0.504), whereas the SON group demonstrated the lowest value (0.194 ± 0.024). The PVP-I and SON+PVP-I groups showed OD600 values of 0.230 ± 0.043 and 0.558 ± 0.263, respectively. The negative control (CTRL−) group showed a background OD600 value of 0.164 ± 0.023.

All intervention groups showed lower residual crystal violet-stained biomass than the untreated positive control. Among the intervention groups, the SON group showed the lowest mean OD600, followed by the PVP-I and SON+PVP-I groups. Notably, the SON+PVP-I group retained a higher mean OD600 than either SON or PVP-I alone, despite the absence of detectable culturable bacteria under the assay conditions.

**Figure 3:**
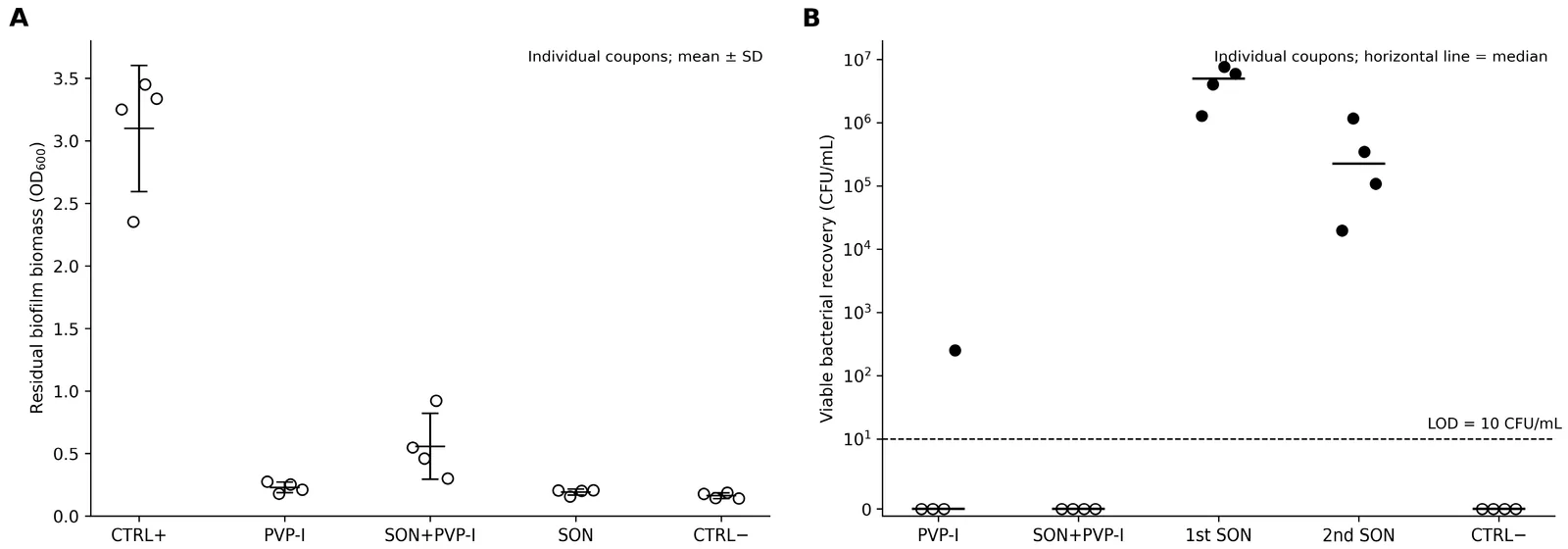
Biofilm biomass and viable bacterial recovery following intervention. (**A**) Residual biofilm biomass measured by crystal violet staining (OD600). (**B**) Viable bacterial recovery expressed as CFU/mL; LOD, limit of detection.

Exploratory statistical analysis demonstrated an overall difference in OD600 values among the five experimental groups (Kruskal–Wallis, H(4) = 16.871, *p* = 0.002). However, none of the pairwise comparisons remained statistically significant after Holm adjustment for multiple comparisons. The SON+PVP-I group also demonstrated greater variability than the SON group (coefficient of variation, 47.2% vs. 12.3%). Individual coupon values are presented in Figure 3A.

### 3.2. Viable Culturable Bacterial Recovery

The pattern of culturable bacterial recovery differed from that observed for residual crystal violet-stained biomass. In the PVP-I group, the recovered bacterial count was 6.25 × 10^1^ CFU/mL (1.80 log10 CFU/mL). No culturable bacteria were detected above the assay detection limit (<10 CFU/mL) in samples from the SON+PVP-I group under the applied culture conditions. No bacterial growth was detected in the CTRL− group.

In the SON group, bacterial recovery following the initial treatment sonication was 4.74 × 10^6^ CFU/mL (6.68 log10 CFU/mL). An additional 4.12 × 10^5^ CFU/mL (5.62 log10 CFU/mL) was recovered following the subsequent sonication step. These measurements represent bacterial recovery at different stages of the sonication workflow and should not be interpreted as a direct reduction in bacterial burden between the two measurements.

Overall, the SON group showed the lowest residual crystal violet-stained biomass, whereas no culturable bacteria were detected above the assay detection limit in the SON+PVP-I group under the applied culture conditions. Because the bacterial recovery procedures were not fully equivalent across the intervention groups, CFU findings were interpreted within the respective treatment workflows rather than as direct quantitative comparisons of total residual viable bacterial burden between groups. Individual CFU values are presented in Figure 3B.

## 4. Discussion

The present study evaluated a low-cost ultrasonic bath sonicator, used alone or in combination with povidone–iodine (PVP-I), for reducing biofilm biomass and culturable bacterial recovery on UHMWPE surfaces in an in vitro periprosthetic joint infection model. The main findings were that: (1) sonication in normal saline resulted in the lowest residual crystal violet-stained biomass; (2) no culturable bacteria were detected in the SON+PVP-I group under the applied culture conditions, corresponding to a bacterial count below the assay detection limit (<10 CFU/mL); and (3) the different patterns observed between residual biomass and culturable bacterial recovery suggest that mechanical biofilm removal and bacterial killing may represent related but distinct aspects of surface decontamination. However, these findings should be interpreted as exploratory because of the small sample size and differences in bacterial recovery procedures among the experimental groups.

Biofilm formation remains one of the major barriers to successful treatment of PJI. The extracellular polymeric substance (EPS) matrix protects microorganisms from host immune responses and limits the penetration and effectiveness of antimicrobial agents, thereby promoting bacterial persistence and treatment failure [4,5,6]. Consequently, effective decontamination strategies may need to address both the physical removal of surface-associated biofilm and the viability of bacteria retained within or released from the biofilm matrix.

In the present study, sonication in normal saline resulted in the lowest residual crystal violet OD600 value. Ultrasound has been proposed to disrupt biofilms through acoustic cavitation, in which oscillation and collapse of microbubbles can generate localized mechanical forces capable of disrupting surface-associated biofilm structures [8,9]. Previous studies have also demonstrated the potential of ultrasound-based approaches for biofilm removal from implant materials. Russo et al. reported that piezoelectric ultrasonic debridement reduced bacterial burden and improved biofilm removal on orthopedic implant materials compared with pulse lavage [11], while Wu et al. demonstrated substantial biofilm reduction using direct-contact low-frequency ultrasound combined with pulse lavage [12]. However, the present study used an indirect ultrasonic bath system, and the acoustic energy and cavitation intensity delivered to the treatment solution were not directly measured. Therefore, cavitation should be considered a plausible mechanism rather than a directly demonstrated explanation for the observed findings.

Recent studies have further suggested that ultrasound-induced changes in biofilm structure may enhance the effectiveness of antimicrobial treatment. Nahum et al. demonstrated that low-frequency ultrasound reduced biofilm mechanical strength and enhanced antibiotic-mediated bacterial inactivation, with mathematical modeling suggesting increased antibiotic diffusivity through the disrupted biofilm [16]. Wu et al. similarly demonstrated ultrasound-mediated disruption of the EPS network and increased susceptibility to antimicrobial effects, although their study employed an ultrasound-responsive nanomaterial system with a distinct chemical mechanism [17]. These findings provide a plausible mechanistic basis for combining ultrasound with antimicrobial treatment; however, whether similar enhancement of PVP-I activity occurred in the present study cannot be determined from the current experimental design.

The relationship between residual biomass and bacterial recovery was not straightforward. Although the SON group showed the lowest residual OD600 value, no culturable bacteria were detected in the SON+PVP-I group, with bacterial recovery below the assay detection limit (<10 CFU/mL). Crystal violet staining measures total surface-associated biomass, including bacterial cells, extracellular matrix components, and cellular debris, and does not distinguish between viable and nonviable bacteria. Therefore, mechanical biofilm removal and bacterial killing should not be expected to produce identical outcomes. The higher residual OD600 observed in the SON+PVP-I group compared with the SON group may therefore reflect residual nonviable biomass or extracellular material rather than persistent viable bacteria. This interpretation is consistent with the different biological targets measured by crystal violet staining and culture-based bacterial recovery. However, because no complementary structural or viability assays, such as scanning electron microscopy, confocal microscopy, or viability staining, were performed, this interpretation remains hypothetical.

In addition, the SON+PVP-I group demonstrated greater variability in OD600 values than the SON group (coefficient of variation, 47.2% vs. 12.3%). Although exploratory statistical analysis demonstrated an overall difference among groups, the pairwise difference between SON and SON+PVP-I did not remain statistically significant after adjustment for multiple comparisons. Therefore, this numerical difference should be interpreted cautiously until confirmed in larger studies.

Sonication has also been widely used to facilitate the recovery of bacteria from biofilm-associated surfaces for microbiological diagnosis [10,18]. The recovery of substantial numbers of culturable bacteria during sonication in the present study is consistent with the ability of ultrasound to release adherent bacteria from surface-associated biofilms. However, the bacterial recovery procedures were not fully equivalent across the experimental groups. Consequently, CFU values should not be interpreted as standardized measurements of total residual viable bacterial burden or used for direct quantitative comparisons between treatment groups. In particular, bacterial counts obtained at different stages of the SON workflow represent sequential bacterial recovery rather than a direct measurement of bacterial killing between sonication steps. This methodological limitation also prevents firm conclusions regarding the relative bactericidal effects of sonication alone and combined treatment.

The absence of detectable culturable bacteria in the SON+PVP-I group suggests that the combination of ultrasound and PVP-I may provide antibacterial activity in addition to mechanical biofilm disruption. Povidone–iodine has broad antimicrobial activity and has demonstrated activity against biofilm-associated microorganisms in vitro [14]. Nevertheless, the present findings should not be interpreted as evidence of complete bacterial eradication. Residual viable bacteria below the culture detection limit or viable but non-culturable bacterial populations cannot be excluded because no orthogonal viability assay was performed. Further studies incorporating complementary viability methods are required to determine whether the combination can achieve more complete bacterial elimination.

An additional finding was that the SON+PVP-I group showed greater residual crystal violet staining than the SON group despite having no detectable culturable bacteria. Another possible explanation is that residual povidone–iodine or PVP-derived material may have influenced the crystal violet assay. Although coupons were washed before crystal violet staining, an abiotic control consisting of sterile coupons exposed to PVP-I was not included. Therefore, potential colorimetric interference or residual surface effects cannot be completely excluded and may have contributed, at least in part, to the higher OD600 values observed in the SON+PVP-I group. The physical properties of the sonication medium, including viscosity, can influence cavitation behavior and energy transmission [19]. However, the viscosity of the treatment solutions, acoustic power delivered to the samples, cavitation intensity, and temperature changes during sonication were not measured in this study. Therefore, any proposed effect of PVP-I on cavitation efficiency remains speculative and requires direct experimental validation.

*Staphylococcus epidermidis* was selected because of its strong biofilm-forming capacity and clinical relevance to implant-associated infection [13]. Eight pilot optimization experiments were performed before the main experiment to establish the biofilm formation protocol on UHMWPE coupons. These experiments identified variability in background crystal violet staining and informed the selection of coupons with background OD600 values <0.5, as well as the final staged incubation protocol. Although this optimization improved the consistency of the experimental model, the findings remain specific to *S. epidermidis* biofilms on UHMWPE under the conditions tested and should not be generalized to other PJI pathogens or implant materials.

The present study provides preliminary proof-of-concept data using a commercially available, low-cost ultrasonic bath rather than a specialized ultrasonic debridement system. This approach may be useful for investigating accessible ultrasound-based decontamination strategies. However, the current experimental setup involved indirect transmission of ultrasonic energy through sealed sample wells, and the actual acoustic energy delivered to the samples was not quantified. Therefore, the findings cannot be directly extrapolated to other ultrasonic devices or treatment configurations, and clinical application should not be inferred from the present results.

Several limitations should be acknowledged. First, the study was exploratory and included only four coupons per group, without a formal sample size calculation, limiting statistical inference. Second, bacterial recovery procedures were not fully standardized across all treatment groups, restricting direct comparisons of CFU outcomes. Third, crystal violet staining measures total biomass rather than bacterial viability, and no complementary structural or viability assay was performed. Fourth, only *S. epidermidis* and a single set of sonication parameters (40 kHz, rated device power of 60 W, and 10 min) were evaluated. The effects of varying PVP-I concentrations, sonication frequencies, powers, and treatment durations therefore remain unknown. Fifth, the actual acoustic energy and cavitation intensity delivered through the indirect sonication setup were not measured, and temperature changes during sonication were not continuously monitored, and their potential contribution to antibacterial activity or biofilm disruption therefore could not be determined. Sixth, potential effects of sonication on UHMWPE surface integrity, including surface roughness or microdamage, were not assessed. Finally, the in vitro model did not reproduce the effects of synovial fluid, host proteins and cells, immune responses, or physiological joint mechanics. Further studies using larger sample sizes, standardized bacterial recovery methods, additional clinically relevant pathogens, complementary biofilm and viability assessments, and evaluation of implant surface integrity are needed before considering translation to clinical decontamination strategies.

## 5. Conclusions

In this exploratory in vitro study, sonication in normal saline resulted in the lowest residual crystal violet-stained biomass on UHMWPE surfaces, whereas no culturable bacteria were detected following combined sonication and povidone–iodine treatment under the applied culture conditions (<10 CFU/mL). These findings suggest that ultrasound-based biofilm disruption and antimicrobial treatment may have complementary roles in surface decontamination. However, differences in bacterial recovery procedures, the small sample size, and the absence of complementary structural and viability assessments limit main conclusions regarding bacterial eradication or treatment superiority. Further studies using standardized recovery methods, larger sample sizes, additional clinically relevant pathogens, and clinically representative models are required before potential clinical application can be considered.

## Figures and Tables

**Table 1:** Summary of pilot experiments performed to optimize *Staphylococcus epidermidis* biofilm formation on UHMWPE coupons.

Pilot	Experimental Conditions Evaluated	Main Observations	Modification or Decision
1	Initial biofilm formation conditions using UHMWPE coupons	Incomplete and inconsistent exposure of coupon surfaces to the bacterial suspension was observed, partly because of coupon positioning and flotation.	Conditions were modified to improve coupon immersion and exposure to the bacterial suspension.
2	Prolonged static biofilm incubation for 7 and 14 days	Prolonged incubation did not consistently distinguish biofilm-associated staining from background staining, with considerable between-coupon variability.	Shorter incubation periods and shaking conditions were evaluated.
3	Biofilm formation using 48-h shaking incubation	Mean OD600 was higher in *S. epidermidis*-exposed coupons than in controls (1.179 vs. 0.823), although substantial between-coupon variability remained.	Staged incubation protocols with separate growth, adhesion, and maturation phases were evaluated.
4	Comparison of two staged protocols: (4.1) 3-h shaking followed by 48-h static incubation; (4.2) 24-h shaking, 3-h static adhesion, followed by 48-h shaking maturation	Both protocols produced higher OD600 values in *S. epidermidis*-exposed coupons than in controls. Protocol 4.2 showed clearer separation between groups. Variable background staining among control coupons was identified and was not attributable to exposure to glucose-containing medium.	Background crystal violet staining of UHMWPE coupons was considered in subsequent experiments.
5	Repeat evaluation of the staged protocols using coupons with background crystal violet OD600 < 0.5	Lower background staining was observed in the control groups. The 24-h growth, 3-h static adhesion, and 48-h shaking protocol produced greater separation between *S. epidermidis*-exposed and control coupons (mean OD600, 1.873 vs. 0.341) than the alternative protocol (0.815 vs. 0.278), although variability remained.	The staged growth–adhesion–maturation protocol was selected for further optimization.
6	Extension of biofilm maturation to 7 days with preliminary evaluation of 7-min sonication at 40 kHz	Robust biofilm-associated staining was observed before treatment. Sonication reduced residual crystal violet staining, although substantial variability remained among *S. epidermidis*-exposed coupons.	Shorter maturation periods were subsequently evaluated.
7	Evaluation of 72-h and 120-h maturation periods followed by 7-min sonication at 40 kHz and bacterial recovery	Viable bacteria were recovered following sonication at both maturation periods. The 72-h model demonstrated low residual crystal violet staining after sonication, whereas the 120-h model showed greater variability and persistent residual biomass.	A 72-h maturation period was favored for subsequent validation.
8	Validation of 72-h and 120-h maturation periods using coupons selected according to a background crystal violet OD600 threshold of <0.5, with two sequential 7-min sonication steps at 40 kHz	In the 72-h model, mean bacterial recovery was 8.74 × 10^6^ CFU/mL after the first sonication and 4.18 × 10^5^ CFU/mL after the second sonication, with residual OD600 approaching the control value (0.220 vs. 0.207). The 120-h model showed greater residual biomass (0.310 vs. 0.130 in controls) and greater variability.	The staged protocol consisting of 24-h growth, 3-h static adhesion, and 72-h shaking maturation at 100 rpm was selected for the main experiment.

## Data Availability

The data used and/or analyzed during the current study are available from the corresponding author.

## Abbreviations

The following abbreviations are used in this manuscript:

PJI	periprosthetic joint infection
UHMWPE	ultra-high-molecular-weight polyethylene
PVP-I	povidone–iodine
SON	sonication only
SON+PVP-I	sonication with povidone–iodine
OD600	optical density at 600 nm
CFU/mL	colony-forming unit counts per milliliter
EPS	extracellular polymeric substance
DAIR	debridement, antibiotics, and implant retention
CTRL−	Negative control
CTRL+	Positive control
TSA	tryptic soy agar
PBS	phosphate-buffered saline
SD	standard deviation
